# Effects of fruits and vegetables on gut microbiota in a mouse model of metabolic syndrome induced by high‐fat diet

**DOI:** 10.1002/fsn3.3114

**Published:** 2022-11-05

**Authors:** Congcong Yu, Cang Guo, Xueying Geng, Yuyang Yao, Junxia Guo, Yanzhen Zhang, Jing Zhang, Shengquan Mi

**Affiliations:** ^1^ College of Biochemical Engineering Beijing Union University Beijing China; ^2^ Changping Laboratory Beijing China; ^3^ Beijing Key Laboratory of Bioactive Substances and Functional Foods Beijing Union University Beijing China

**Keywords:** C57BL/6N mice, fruits and vegetables, gut microbiota, metabolic syndrome

## Abstract

The aim of this study was to evaluate the effect of fruit and vegetable intake on gut microbiota using a mouse model of metabolic syndrome (MS) induced by a high‐fat diet. Forty‐eight male mice were randomly divided into four groups, control group (C), high‐fat diet‐fed model group (H), high fat plus low intake of fruits and vegetables diet‐fed group (H.LFV), high fat plus high intake of fruits and vegetables diet‐fed group (H.HFV), and each group were fed for 60 days. During the experiment, mouse body weights were recorded and fecal samples were collected. Cetyltrimethyl ammonium bromide (CTAB) method was used to extract fecal bacterial DNA, and the purity and concentration of the DNA were detected by electrophoresis. DNA samples underwent PCR amplification (primers in 16 S V4 (515F and 806R)). Raw sequencing data were processed, and sample complexity and multiple‐sample comparisons were investigated. Mouse organ coefficient, serum lipid levels, fecal TC (total cholesterol) and TBA (total bile acid) levels, and hepatic glutathione and malondialdehyde levels were determined. Compared to the H group, the fecal TC and TBA levels decreased significantly in the H.HFV group (*p* < .05), and hepatic glutathione and malondialdehyde levels decreased significantly in both H.LFV and H.HFV groups (*p* < .05). Decreased abundance of Firmicutes, Burkholderiales, Syntrophomonas, and Pseudomonadales in gut microbiota was observed in H.LFV and H.HFV groups compared to the H group. The Anosim results showed significant differences in pairwise comparison between groups. The linear discriminant analysis effect size (LEfSe) results showed that k_bacteria not only exhibited statistically differences between H and C groups but also among H.LFV, H.LFV, and H groups, and hence, could be used as a biomarker between groups. To sum up, fruit and vegetable powder could increase the fecal excretion of TC and TBA, and the antioxidant capacity in C57BL/6N mice. Meanwhile, the mechanism that fruit and vegetable powder could prevent MS in C57BL/6N mice was related to the decreased abundance of gut microbiota, including Firmicutes, Syntrophomonadales, and Pseudomonadales. Hence, fruit and vegetable powder could be used as a recommended food to regulate gut microbiota and prevent the occurrence of MS‐related diseases.

## Graphical abstract

The aim of this study was to evaluate the effect of fruit and vegetable intake on gut microbiota using a mouse model of metabolic syndrome (MS) induced by high‐fat diet. our research shows that continuous intake of a certain amount of fruits and vegetables can significantly inhibit the increase of physical fitness, normalize the liver lipid accumulation in mice with metabolic syndrome, and reduce the risk of metabolic diseases; increase C57BL/ TC and TBA excretion levels in feces of 6N mice; improvement of liver antioxidant levels in C57BL/6N mice. In addition, fruit and vegetable intake not only promoted the abundance of Firmicutes, but also inhibited Bacteroidetes, thereby increasing the diversity of gut microbiota in mice. Therefore, fruits and vegetables have a good prebiotic effect in the regulation of intestinal flora, and have the potential to prevent and improve metabolic syndrome and other related diseases.

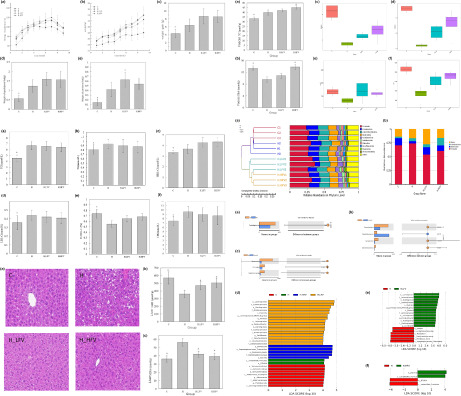

## 1 INTRODUCTION

Metabolic syndrome (MS) is a pathological condition in which the metabolism of proteins, fats, and carbohydrates is disturbed, and manifests with multiple symptoms such as abdominal obesity and/or overweight, hypertension, type 2 diabetes mellitus (T2DM), insulin resistance, and/or impaired glucose tolerance, dyslipidemia, microalbuminuria, hyperuricemia, fatty liver, and hyperhomocysteinemia, among others (Fahed et al., 2022). Currently, MS is a major clinical challenge worldwide. By 2017, more than 1 billion people worldwide had MS, and approximately 33.3% of Americans and 15.5% of Chinese had MS (Saklayen, 2018). Advanced age, overweight/obesity, smoking, excessive alcohol consumption, insufficient physical activity, and unhealthy diet have been found to be risk factors for MS (Castro‐Barquero et al., 2020; Tran et al., 2017; Wadden et al., 2012; Xi et al., 2013). Among them, diet is an important factor (Cheng et al., 2022). Research confirms that the Mediterranean diet is characterized by high consumption of fruits, vegetables, nuts, olive oil, and fish, and low intake of saturated fat, red and processed meat, refined carbohydrates, and full‐fat dairy products (Merra et al., 2020), and was negatively correlated with MS (Godos et al., 2017; Viscogliosi et al., 2013). A Korean study also found that, after controlling for confounders, a dietary pattern with a higher intake of alcohol and meat was associated with an increased risk of MS, while a dietary pattern with a higher intake of fish, grains, and vegetables was associated with MS risk and negative correlation (Kim & Jo, 2011). Therefore, dietary therapy has attracted the attention and heated discussion of biochemical researchers in the potential human health problem‐metabolic syndrome MS. At present, people pay more attention to a healthy diet, a diet rich in fruits and vegetables, and researchers are also committed to discovering its potential mechanism of action.

The gut microbiota (GM) plays an important role in the pathogenesis of MS. When the balance between GM and the host immune system is disrupted to a certain extent, bacterial fragments are displaced and “metabolic endotoxemia” occurs, leading to systemic inflammation, liver tissue lesions, and insulin resistance (Festi et al., 2014). Among the many factors involved, whether endogenous to the host or exogenous to the host, diet is a key factor in determining the structure and function of transgenes (Zmora et al., 2019). On the one hand, dietary fiber has an effect on the composition of GM in the diet, thereby reducing the risk of MS. Zhao conducted a clinical randomized controlled trial in 43 patients with type 2 diabetes. Before the intervention, the intestinal flora of the participants was transplanted. After the intervention, the germ‐free mice in the experimental group and the control group after the intervention were different from those before the transplantation. Compared with mice with gut microbiota, experimental and control mice showed better satisfaction with metabolic parameters after transplantation intervention. These results confirm that dietary fiber affects the gut microbiota to improve MS (Zhao et al., 2018). On the other hand, dietary fiber in the diet cannot be absorbed by the upper gastrointestinal tract, but is fermented by GM into various metabolites, including short‐chain fatty acids (SCFA) such as acetate, propionate, and butyrate (Cani, 2019). Increased SCFA reduces pH in the gut, thereby inhibiting the growth of Enterobacteriaceae, which produce lipopolysaccharide (LPS) (Delzenne et al., 2013; Everard et al., 2013), which is a common bacterial antigen that causes low‐grade inflammation leading to the development of MS (Conlon & Bird, 2014; He et al., 2015). Fruits and vegetables play an important role in the human diet and are rich in dietary fiber, which can help regulate the homeostasis of the gut microbiota, thereby reducing the risk of MS. However, due to the population‐based sample study, the research environment is difficult to strictly control, and its influencing factors are more complex. Therefore, we plan to explore the effects of vegetables and fruits on the gut–liver axis at the animal level based on the results of epidemiological studies.

Fresh fruits and vegetables are an important part of a balanced diet. In the Dietary Guidelines for Chinese Residents (2016), fresh fruits and vegetables are on the second level of the food guideline pyramid. The recommended intake of vegetables is 300–500 g/day, and the recommended intake of fruits is 200–350 g/day (Wang et al., 2016). There are some epidemiological studies on the association of fruit and vegetable intake with MS risk, but the results are inconsistent. A cross‐sectional study conducted in Jilin Province, China, in 2012 showed that consumption of fresh fruit more than twice a week was associated with a lower risk of MS, whereas high vegetable intake was not associated with MS incidence (Wu et al., 2016). However, Zhang performed a meta‐analysis of the association between fruit and vegetable consumption and MS, using data from 26 observational studies published up to September 2017. Results showed that vegetable and/or fruit consumption was inversely associated with MS (Zhang & Zhang, 2018). Thus, current epidemiological investigations of MS and other diseases suggest an indeterminate association between fruit and vegetable consumption and MS incidence, and the mechanism of action remains to be determined in future studies. Compared with epidemiological experiments, animal experiments can effectively control uncertain factors, accurately grasp dietary intake, and save time and effort. Therefore, on the basis of epidemiological experimental data, this study chose to use animal experiments to confirm the relationship between fruits and vegetables and MS. In our study, a high‐fat diet‐induced metabolic syndrome model in C57BL/6 N mice was established to investigate the possible mechanism of action between fruit and vegetable intake and MS. We aimed to preliminarily explore the effect of dietary intake on gut microbiota and provide a theoretical basis for a reasonable diet.

## 2 MATERIALS AND METHODS

### 2.1 Animals, materials, and reagents

Specific pathogen‐free (SPF) male C57BL/6 mice, 6–8 weeks of age, were purchased from Speifu Biotechnology Co., Ltd. (SCXK (Jing) 2019‐0010). The body weight was (16 ± 1) g. Mice were housed in single cages in the SPF animal room of Beijing Union University with free access to food and water. The conditions of the animal room were as follows: room temperature was 22°C ± 2°C, relative humidity was 55 ± 5%, and the lights were switched on and off for 12 h. The standard chow diet (MD12031) and high‐fat diet (MD12032) were obtained from Medison Bio‐Pharmaceutical Co., Ltd. (Jiangsu, China). Fresh fruits and vegetables were purchased from Yonghui Supermarket (Beijing, China). Assay kits for TG, TC, HDL‐C, LDL‐C, FBG, TBA, GSH, and MDA were purchased from Jiancheng Bioengineering Institute; Science and Technology Co., Ltd. (Nanjing, China).

### 2.2 Preparation of fruit and vegetable powder

The formula of the standard chow diet and high‐fat diet is shown in Table 1. Fruit and vegetable powder consisted of five different types of vegetables and five different types of fruits, and its formula was determined according to the recommended daily intake of vegetables and fruits (vegetables 500 g, fruits 250 g) in the Dietary Guidelines for Chinese Residents (2016) (Table 2). Fresh fruits and vegetables were washed, peeled, cored, air‐dried, beaten, dried, crushed, and passed through a sieve with a nominal mesh aperture of 1.00 mm. According to the Dietary Guidelines for Chinese Residents (2016), a 60 kg adult should consume 500 g of vegetables and 250 g of fruits daily. Since the metabolism of mice is 10 times of humans, a mouse should eat 83.2 g/kg of vegetables and 41.6 g/kg of fruits daily, and the total intake of fruits and vegetables should be around 125 g/kg. Preliminary experiments showed that 1 kg of fresh fruits and vegetables could be dried into about 100 g of fruit and vegetable powder. The daily intake of mice was 120 g/kg, so it was finally determined that the high‐fat plus high intake of fruits and vegetables diet group was supplemented with 10% fruit and vegetable powder, and the high‐fat plus low intake of fruits and vegetables diet group was supplemented with 5% fruit and vegetable powder. The powder and diet was prepared by Medison Biomedical Co., Ltd (Jiangsu, China).

**TABLE 1:** Animal feed formulations

Components proportions	Standard chow diet	High‐fat diet	High‐fat plus low intake of fruits and vegetables diet	High‐fat plus high intake of fruits and vegetables diet
Casein (%)	18.96	23.31	23.31	23.31
Corn starch (%)	42.86	8.51	7.45	6.4
Maltodextrin (%)	7.11	11.65	10.2	8.76
Sucrose (%)	16.38	20.14	17.64	15.14
Cellulose (%)	4.74	5.83	5.83	5.83
Soybean oil (%)	2.37	2.91	2.91	2.91
Lard (%)	1.9	20.68	20.68	20.68
Fruit and vegetable powder (%)	0	0	5	1
Minerals and vitamins (%)	5.68	6.97	6.97	6.97
Total (%)	100	100	100	100

**TABLE 2:** Formula of fruit and vegetable powder

Categories	Types	Proportion (%)
Vegetables	Chinese cabbage	26.67
	Tomato	13.33
	Cucumber	13.33
	Carrot	6.67
	Cauliflower	6.67
Fruits	Apple	13.33
	Pear	6.67
	Watermelon	5
	Winter jujube	5
	Grape	3.33

### 2.3 Determination of crude fiber content in fruit and vegetable powder

Determination of crude fiber used the semi‐automatic operation method in “Feeding stuffs—Determination of crude fiber content—Method with intermediate filtration” (GB/T 6434‐2006) as a reference. Approximately 1 g sample was added into a glass crucible and the weight was weighed with an accuracy of 0.1 mg and recorded as M0. The glass crucible was then connected to a digestion cylinder and 30 ml of petroleum ether was added. The sample was washed for 3 min and the dry residue underwent suction drying after washing. The process was repeated three times. Then, 150 ml of boiling sulfuric acid solution and a few drops of the antifoaming agent were added to the sample and were kept vigorously boiling for 30 min, and the dry residue underwent suction drying after acid elution. Then, 30 ml of hot water was added and the solution was kept vigorously boiling for 3 min. The process was repeated at least three times and the dry residue underwent suction drying after washing to make sure the solution was neutral. Then, 150 ml of boiling potassium hydroxide solution and a few drops of anti‐foaming agent were added and the solution was kept vigorously boiling for 30 min, and the dry residue underwent suction drying after alkaline elution. Then, 30 ml of hot water was added and the solution was kept vigorously boiling for 3 min. The process was repeated at least three times and the dry residue underwent suction drying after washing to make sure the solution was neutral. The glass crucible and its contents were put in a drying oven at 130°C for over 2 h. After cooling in a desiccator, it was weighed and recorded as M1. The glass crucible and its contents were placed in a muffle furnace (500 ± 25) °C for ashing. Until the difference between two consecutive weighs did not exceed 2 mg after cooling, the final weight was recorded as *M*2. Crude fiber content (%) = 100 × (*M*1 − *M*2)/*M*0.

### 2.4 Grouping of experimental animals

After adaptive feeding with a standard chow diet for 1 week, the mice were randomly divided into four groups and fed with a standard chow diet (C group), high‐fat diet (H group), high fat plus low intake of fruits and vegetables diet (H.LFV group), and high fat plus high intake of fruits and vegetables diet (H.HFV group), respectively. Mice in each group had free access to their corresponding feed and purified water. They were weighed every week and the amount of feed intakes was recorded. All mice were killed for autopsy after 9 weeks.

### 2.5 Determination of the serum biochemical indicators

Orbital sinus blood was collected from mice and left at room temperature for 30 min. Then, the blood was centrifuged at 3000 r/min for 10 min, the supernatant was aspirated, and stored at −80°C for later use. Blood lipids were measured using commercial kits according to the manufacturer's instructions. Briefly, the determination of TG and TC adopted the single‐reagent GPO‐PAP method. The analysis of HDL‐C and LDL‐C adopted the double‐reagent direct method. FBG assay adopted the glucose oxidase method. All parameters were measured using a microplate reader (Thermo Scientific Company, USA).

### 2.6 Histopathological examination of liver sections

A predefined position in the right lobe of the liver was fixed in formalin solution for 48 h. The sample then underwent dehydration in gradient alcohol solution, transparent with xylene solution, dipping in wax, embedding, trimming the wax block, sectioning, dewaxing, staining, dehydration, and mounting. Sections were observed with 40x magnification.

### 2.7 Determination of hepatic GSH and MDA levels

Hepatic GSH and MDA levels were determined using commercial kits according to the manufacturer's instructions. All assays used the TBA method and were performed on a spectrophotometer (Shanghai Unico Instrument Co., Ltd., Shanghai, China).

### 2.8 Determination of fecal TC and TBA levels

The feces of the mice were collected within 1 week and placed in a centrifuge tube for use. After freeze‐drying, 0.50 g feces were added with five times normal saline and kept still for 2 h. Then, the mixture was centrifuged at 5000 r/min for 10 min, and the supernatant was used for analysis. Fecal TC and TBA levels were determined according to the instruction of the kits. The analytical method adopted the single‐reagent GPO‐PAP method and a microplate reader was used for determination (Thermo Scientific Company, USA).

### 2.9 Fecal bacterial DNA extraction and 16 S rDNA sequencing

Four mice were randomly selected from each group to collect fresh feces at the same time, and gut microbiota was determined. The fecal bacterial DNA was extracted by cetyltrimethyl ammonium bromide (CTAB) method. The purity and concentration of DNA were detected by electrophoresis. DNA samples then underwent PCR amplification (primers in 16 S V4 (515F and 806R)). Raw sequencing data were processed, and sample complexity and multiple‐sample comparisons were investigated.

### 2.10 Statistical analysis of data

Data were processed using Statistical Package for Social Science (SPSS) 19.0. The comparison of two‐factor repeated measures was analyzed by one‐way ANOVA. Multiple comparisons were analyzed by Dunnett's *t*‐test. *p* < .05 was considered statistically significant.

## 3 RESULTS

### 3.1 Determination of crude fiber content and percentage of energy from nutrients in different diets

As shown in Table 3, the addition of fruit and vegetable powder did not change protein energy, fat energy, carbohydrate energy, and total energy, but only the crude fiber content changed in each group. High fat plus high intake of fruits and vegetables diet (H.LFV) had the highest crude fiber content, accounting for 6.58% and the higher‐fat diet group increased by 0.75%.

**TABLE 3:** Crude fiber content and percentage of energy from nutrients in different diets

Category	Standard chow diet (C)	High‐fat diet (H)	High‐fat plus low intake of fruits and vegetables diet (H.LFV)	High‐fat plus high intake of fruits and vegetables diet (H.HFV)
Crude fiber content (%)	4.74	5.83	6.2	6.58
Percentage of energy from protein (%)	20	20	20	20
Percentage of energy from fat (%)	10	45	45	45
Percentage of energy from carbohydrate (%)	70	35	35	35
Total energy (kcal/g)	3.85	4.73	4.73	4.73

### 3.2 Effect of fruits and vegetables on energy intake and body weight of mice

**FIGURE 1:**
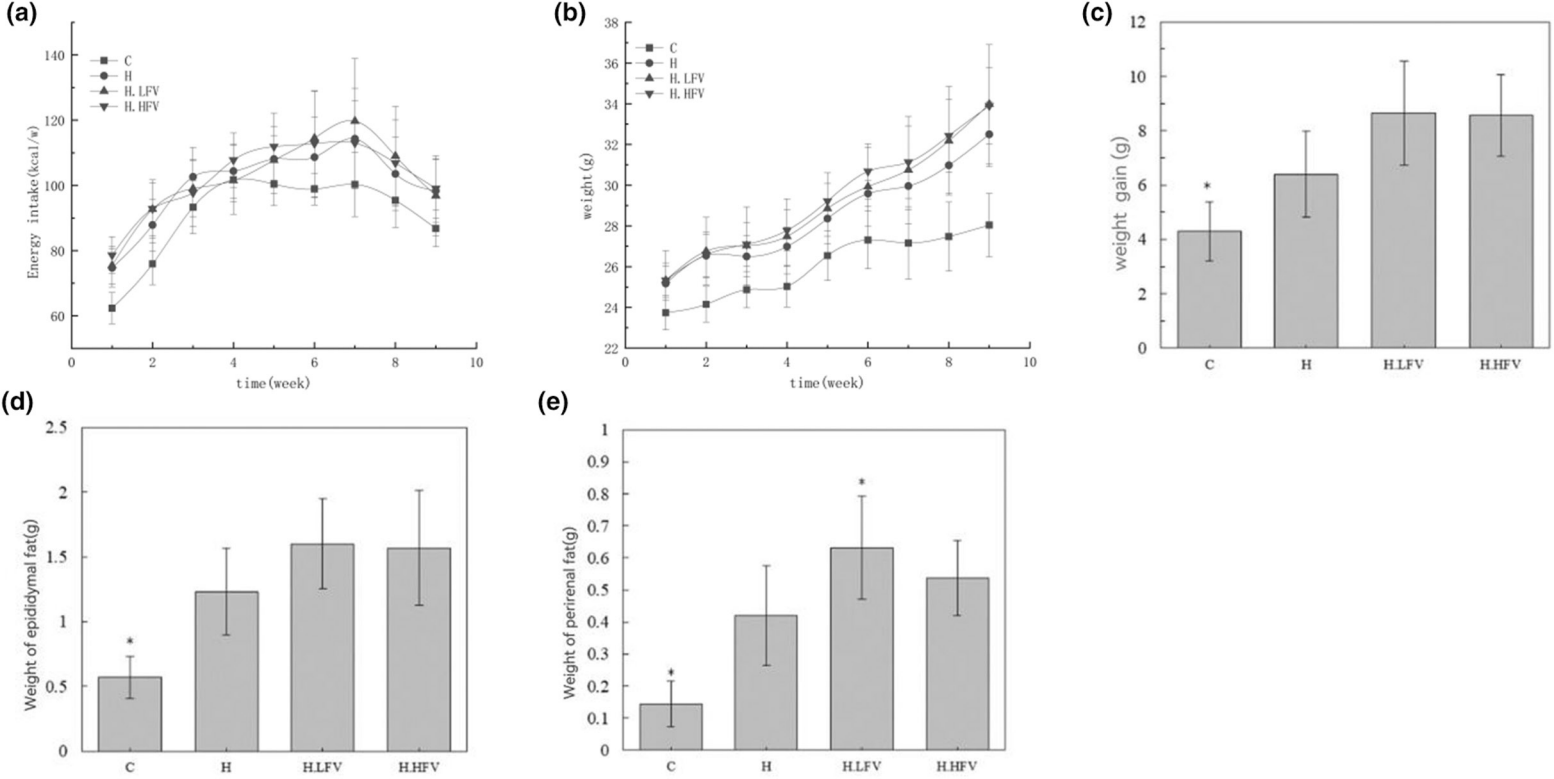
Effects of vegetable and fruit intake on energy intake (a), weight (b), weight gain (c), epididymal fat (d), and perirenal fat (e) in high‐fat diet C57BL/6N mice; *p < .05 compared to H group.

The energy intake level of mice in each group fluctuated to a certain extent during the experimental period, and the energy intake demand first increased and then decreased with the change in the growth cycle of mice. However, there were no statistical differences in energy intake between different groups (*p* > .05) (Figure 1a), indicating that feeding fruits and vegetables to mice with high‐fat diet‐induced MS did not cause insufficient energy intake.

During the experiment, the weight gain of mice in the H group was higher than that in the C group, and the difference was statistically significant (*p* < .05). The weight gain of mice in the H.LFV group and H.HFV group were higher than that in the H group, but the differences were not statistically significant (*p* > .05) (Figure 1b,c). The results suggested that fruit and vegetable intake did not prevent weight gain in mice with high‐fat diet‐induced MS, but it also did not make them gain extra weight. This might be due to the fact that fruits and vegetables contain both sugars and cellulose, which acted together to reach a balance between gaining and losing weight.

Comparing the weight of epididymal fat and perirenal fat in different groups of mice, the results showed that the weight of epididymal fat and perirenal fat in group H were higher than those in group C, and the difference was statistically significant (*p* < .05). The perirenal fat weight in the H.HFV group was higher than that in the H group, and the difference was statistically significant (*p* < .05); the epididymal fat weight and the perirenal fat weight in the H.HFV group were higher than those in the H group, but the difference was not statistically significant (*p* < .05) (Figure 1d,e).

### 3.3 Effect of fruits and vegetables on serum biochemical parameters in mice

**FIGURE 2:**
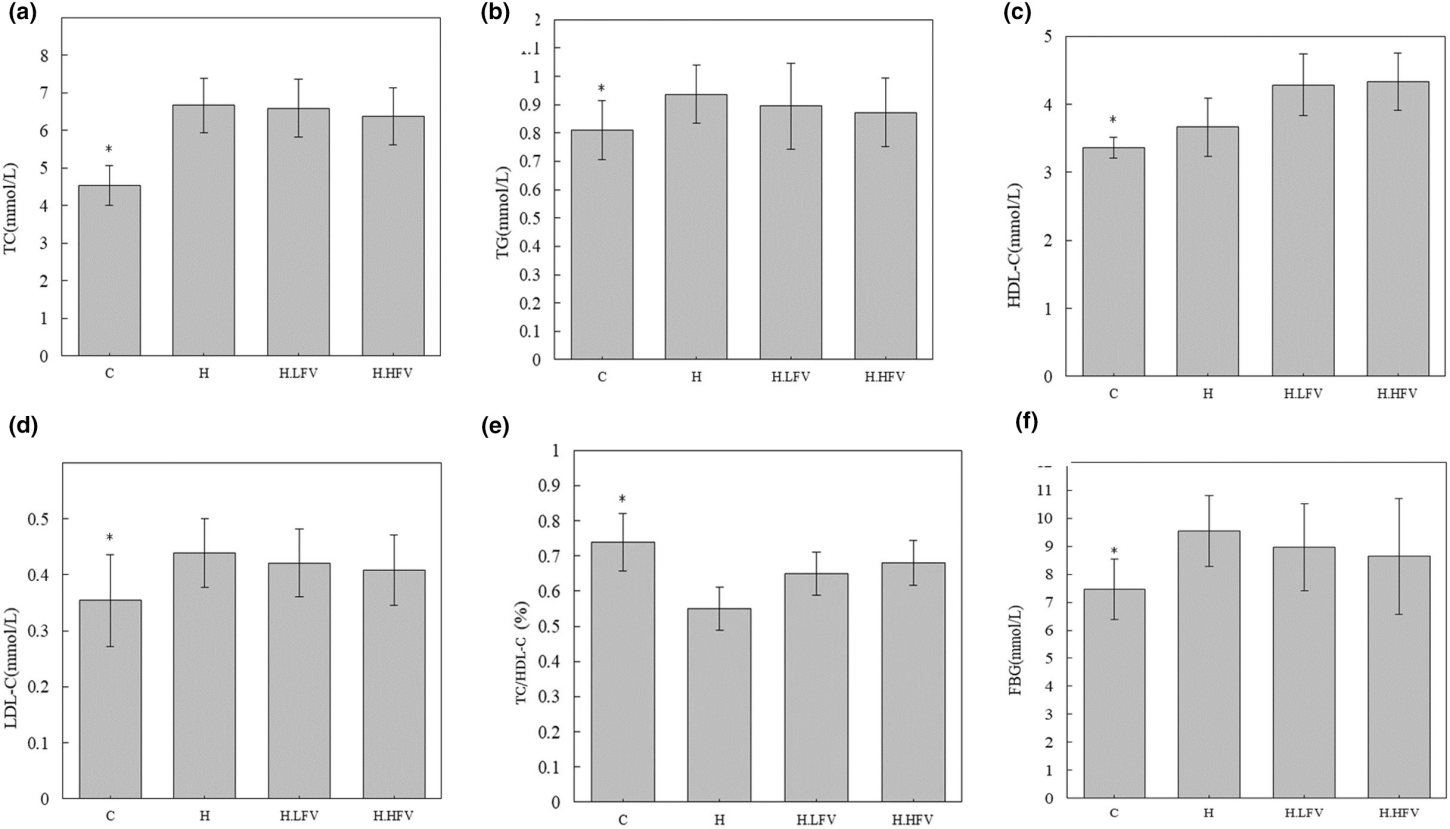
Effects of vegetable and fruit intake on TC (a), TG (b), HDL‐C (c), LDL‐C (d), TC/HDL‐C (e), and FBG (f) in high‐fat diet C57BL/6N mice; *p < .05 compared to H group.

Serum levels of TC, TG, HDL‐C, LDL‐C, TC/HDL‐C, and FBG in different groups were shown in Figure 2. The results showed that TC, TG, HDL‐C, LDL‐C, and FBG in the H group were significantly higher than those in the C group (*p* < .05). TC/HDL‐C ratio in the H group was significantly lower than that in the C group (*p* < .05). Serum levels of TC, TG, LDL‐C, and FBG in H.LFV and H.HFV groups were lower than those in the H group. However, the differences were not statistically significant (*p* > .05). HDL‐C and TC/HDL‐C ratios in the H.LFV group and H.HFV group were higher than those in the H group, but they were not statistically significant (*p* > .05).

### 3.4 Effect of fruits and vegetables on liver pathology in mice

**FIGURE 3:**
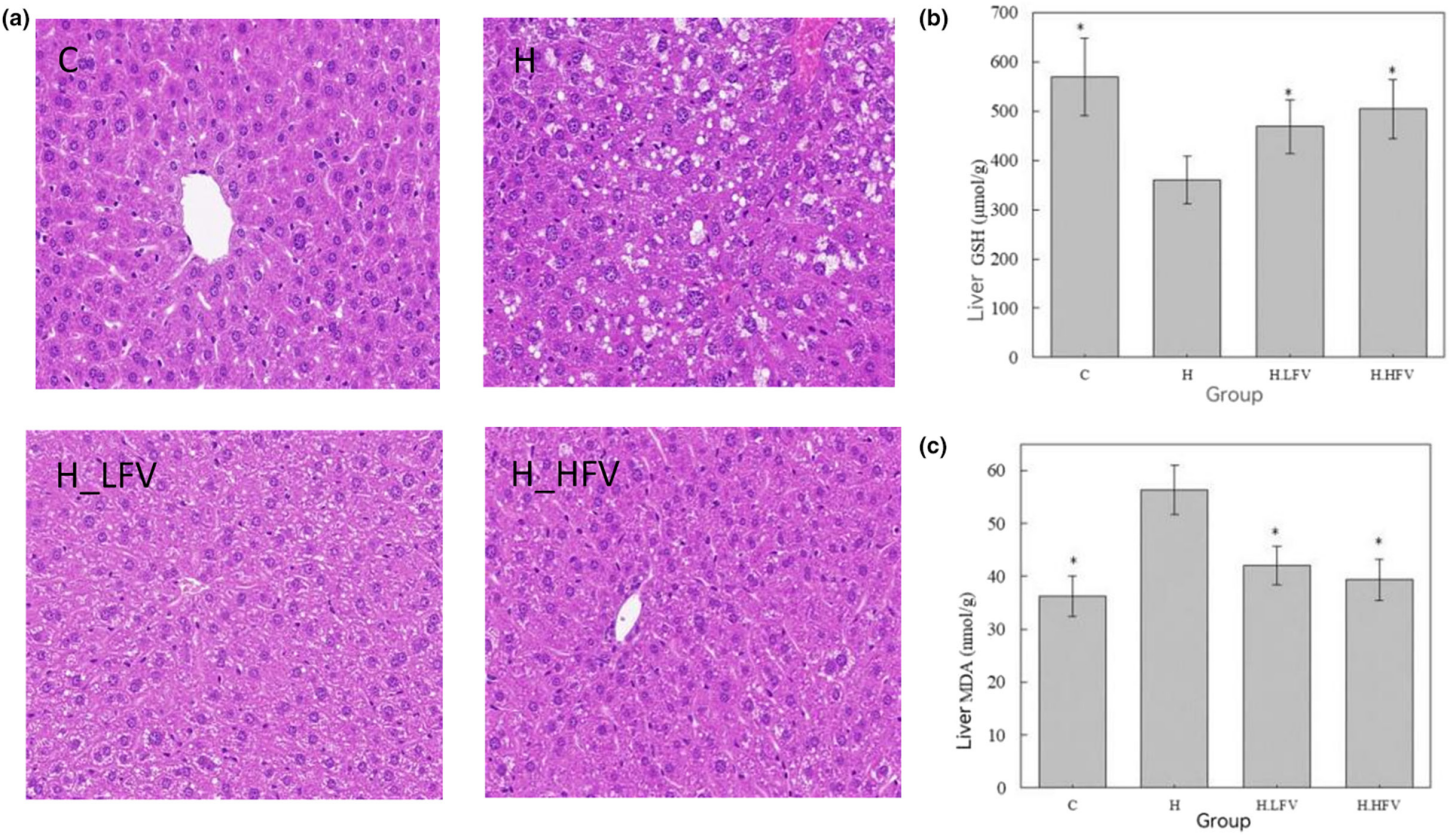
(a) HE staining of liver tissue sections (40); (b,c) effect of vegetable and fruit intake on hepatic GSH and MDA in C57BL/6N mice; *p < .05 compared to H group.

As shown in Figure 3a, the liver cells in the C group were neatly arranged and polygonal in shape. The nucleus was large and located in the central region of the cell, and no obvious fat‐filled vacuoles were found in it. In H group, the liver cells were arranged in disorder and the nucleus deviated from the central region of the cell, and multiple vacuoles could be observed. Compared with the H group, the shape of liver cells in the H.LFV group was improved, the arrangement was more orderly, and the number of fat‐filled vacuoles decreased. In H.HFV group, the liver cell morphology was significantly improved, and the number of fat‐filled vacuoles was significantly reduced compared to H group.

### 3.5 Effect of fruits and vegetables on hepatic GSH and MDA levels in mice

**FIGURE 4:**
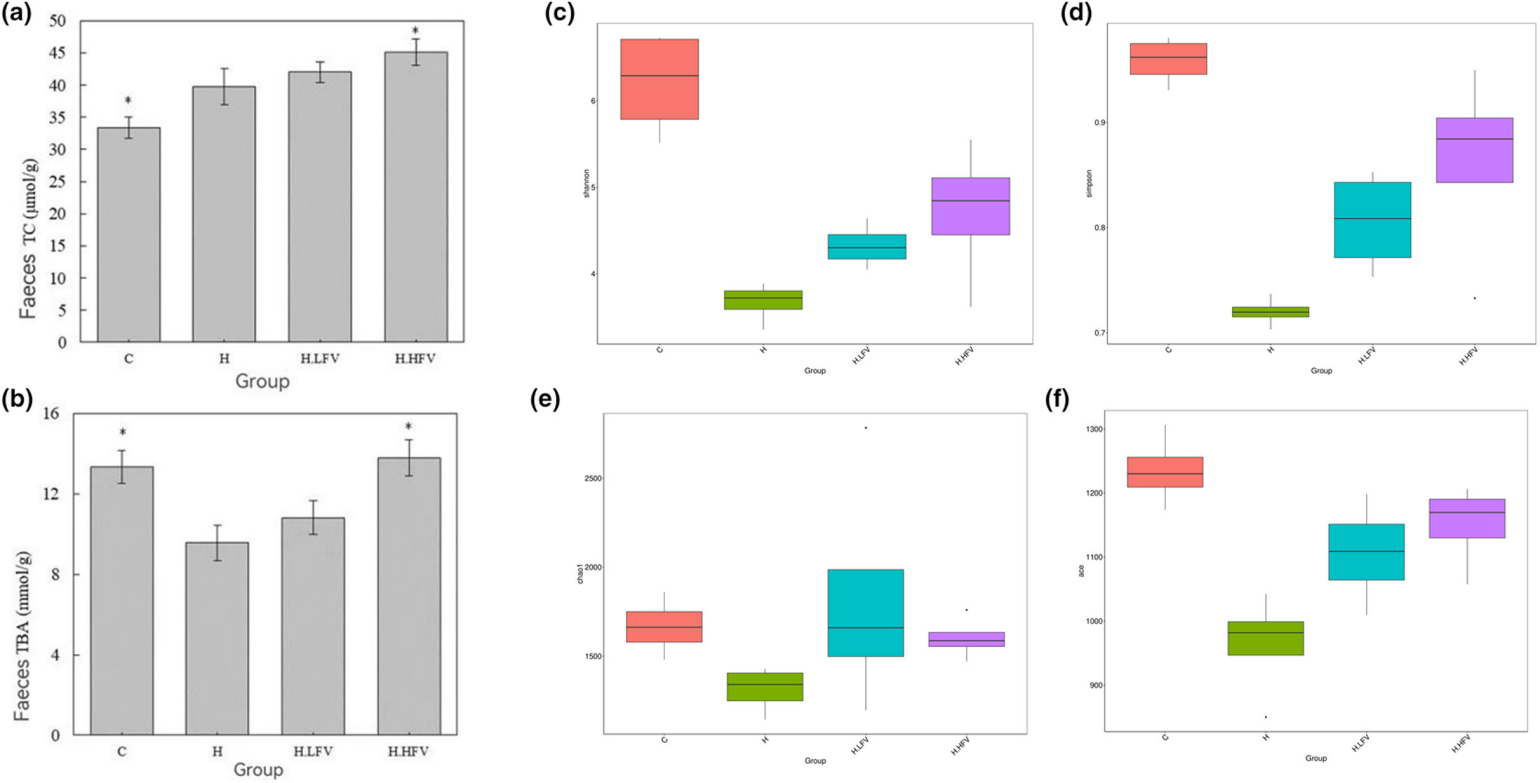
Effect of vegetable and fruit intake on fecal TC and TBA in C57BL/6N mice (a,b); diversity analysis of a group of Shannon, Simpson, Chao1, and ACE (alpha) (c–f); *p < .05 compared to H group.

One‐way ANOVA and Dunnett's *t*‐test for multiple comparisons (H group was used as the reference group) were used to compare hepatic GSH and MDA levels in mice of different groups. The results showed that hepatic GSH levels of C, H.LFV, and H.HFV groups were statistically significantly higher than that of H group (*p* < .05) (Figure 4a). The hepatic MDA levels in C, H.LFV and H.HFV groups were statistically significantly lower than that of H group (*p* < .05) (Figure 3b,c).

### 3.6 Effect of fruits and vegetables on fecal TC and TBA levels in mice

**FIGURE 5:**
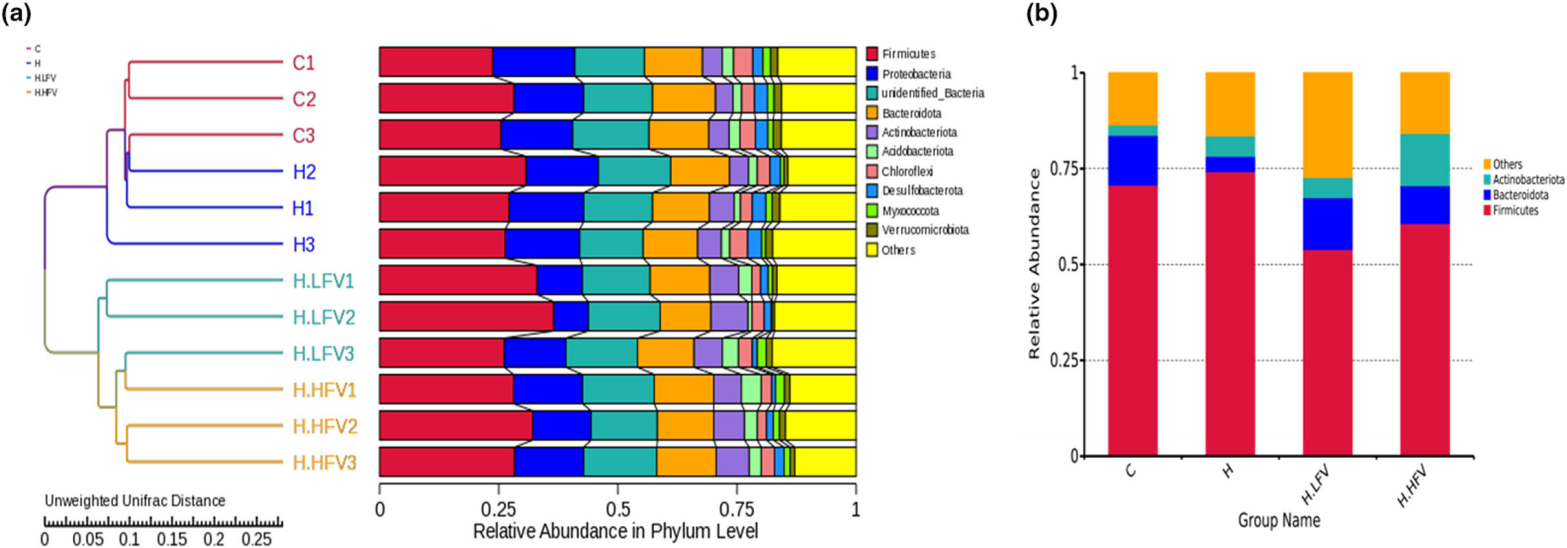
The UPGMA clustering of each group was performed based on the unweighted Unifrac distance (a); bar chart of phylum‐level species abundance (b).

One‐way ANOVA and Dunnett's *t* test were used (H was used as the reference group) to compare fecal TC and TBA levels of mice in different groups. When compared to H group, fecal TC levels were statistically significantly lower in C group, and statistically significantly higher in H.HFV group (*p* < .05) (Figure 5a). Fecal TBA levels in C and H.HFV group were higher than that in H group, and the difference was statistically significant (*p* < .05); as shown in Figure 4a,b.

### 3.7 Effect of fruits and vegetables on the diversity of gut microbiota in mice

The Shannon index and Simpson index reflect community diversity and evenness. The higher the index, the higher the community diversity and the more even the species distribution. As shown in Figure 4c–f, the Shannon index and Simpson index of H group were significantly lower than that of C group (*p* < .05). The Simpson index in H.LFV and H.HFV group was significantly higher than that of H group (*p* < .05), indicating that fruits and vegetables could increase the diversity of gut microbiota in mice with high‐fat diet‐induced MS.

Chao1 and ACE indices reflect the number of species in the community, and the larger the index, the greater the number. The Chao1 and ACE indices of group H were lower than that of group C, but the differences were not statistically significant (*p* > .05). The ACE index of H.LFV group and H.HFV group were higher than those of group H, but the difference was not statistically significant (*p* > .05), suggesting fruits and vegetables had no obvious effect on the number of species of gut microbiota in mice with MS induced by high‐fat diet.

### 3.8 Effect of fruits and vegetables on the composition of gut microbiota in mice

In order to study the similarity between different samples, the unweighted pair group method with arithmetic mean (UPGMA) method was used to construct the clustering tree of the samples. As shown in Figure 5a, when the threshold was set to 0.05, the H and C groups fell into one category, and the H.LFV and H.HFV groups were clustered into another category.

As shown in Figure 5b, at the phylum level, the two species with the highest relative abundance were Firmicutes and Bacterioidota. The microbiota abundance changed with the addition of fruits and vegetables. Compared with the H group, the relative abundance of Firmicutes in the H.LFV and H.HFV group decreased, while the relative abundance of Bacteroidetes increased.

According to previous studies, the ratio of Firmicutes/Bacteroidetes (F/B ratio) can reflect the balance of intestinal microecology and is a typical parameter of the health status of organisms. F/B ratio in H group (18.66) was significantly higher than that in C group (5.46) (*p* < .05). H.LFV and H.HFV groups exhibited significantly lower F/B ratio compared to H group (4.00, 6.15, and 18.66, respectively) (*p* < .05). These results indicated that high‐fat diet could induce gut microbiota dysbiosis in mice, and the intake of fruits and vegetables could possibly modulate the composition of the gut microbiota at the phylum level.

### 3.9 Analysis of differences in gut microbiota compositions in mice caused by the intake of fruits and vegetables at the phylum, class, order, family, and genus levels

**FIGURE 6:**
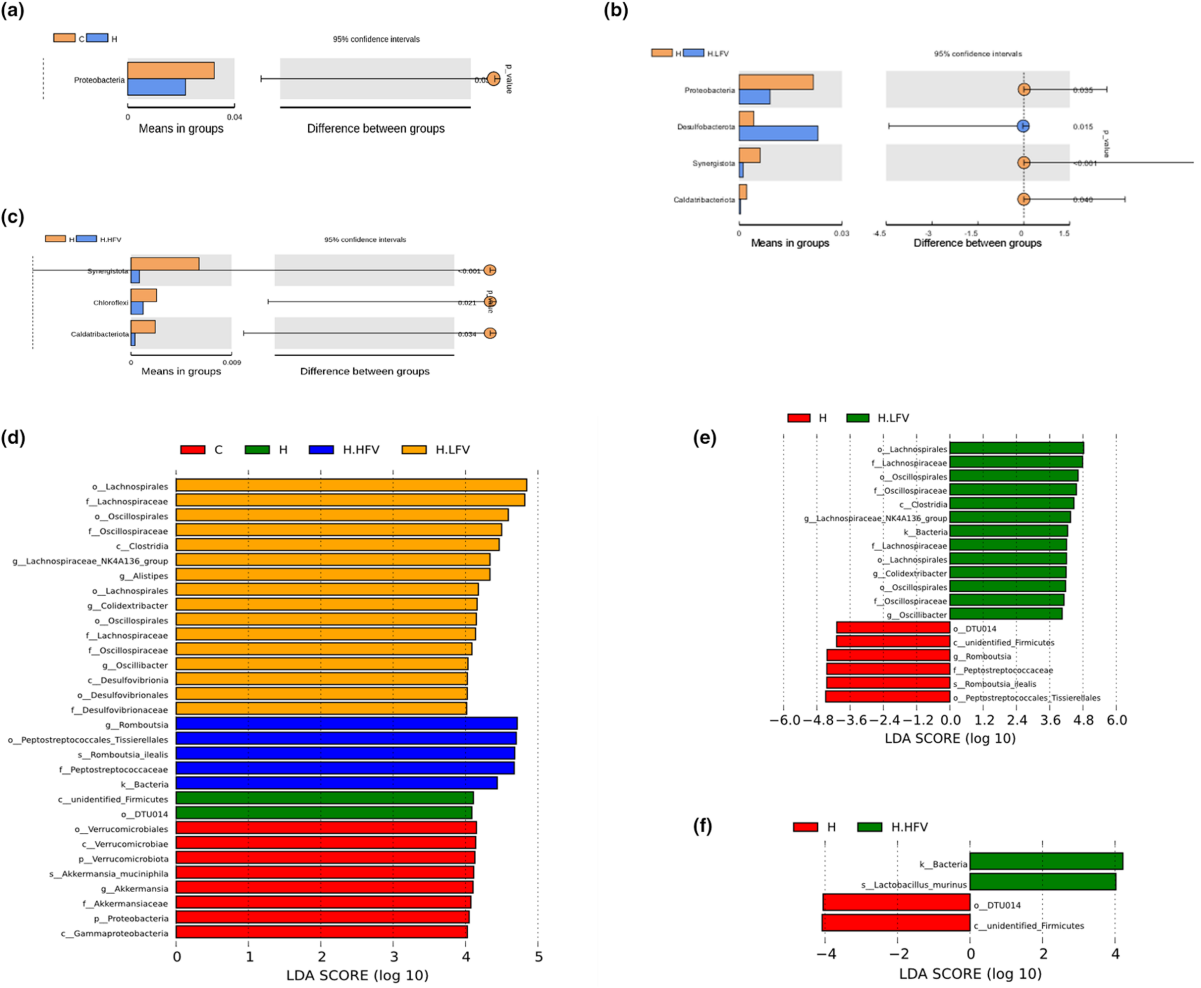
Species with significant differences between the groups (a–c: Door level; d–f: Distribution of LDA values).

As shown in Figure 6a–c, at the phylum level, compared to H group, the abundance of Proteobacteria increased significantly in C group; the abundance of Proteobacteria, Synergistota, and Caldatribacteriota decreased significantly in H.LFV group, while the abundance of Desulfobacterota increased; the abundance of Synergistota, Chloroflexi, and Caldarina decreased significantly in H.HFV group. Species with significant differences at five levels among different diet‐fed groups are listed in Table 4.

**TABLE 4:** Significant differences in abundance among different diet‐fed groups at the phylum, class, order, family, and genus levels

Level	Reference group	Groups compared to reference	Species with significantly decreased abundance	Species with significantly increased abundance
Class	H	C	Gammaproteobacteria	Syntrophomonadia
	H.LFV	H	Gammaproteobacteria, Syntrophomonadia, Synergistia, Bacterodia, Caldatribacteriia, and Anaerolineae	Desulfovibrionia
	H.HFV	H	Syntrophomonadia, Synergistia, Caldatribacteriia, and Anaerolineae	Clostridia
Order	H	C	Burkholderiales, Chitinophagales, and Cellvibrionales	Syntrophomonadales
	H.LFV	H	Burkholderiales, Syntrophomonadales, Synergistales, Caldatribacteriales, Pseudomonadales, Bacteroidales, and Anaerolineales	Desulfovibrionales
	H.HFV	H	Burkholderiales, Syntrophomonadales, Synergistales, Caldatribacteriales, Pseudomonadales, Bacteroidales, and Anaerolineales	Oscillospirales, Lachnospirales, and Monoglobales
Family	H	C	Comamonadaceae, Rhodocyclaceae, Spongiibacteraceae, and Saprospiraceae	Syntrophomonadaceae
	H.LFV	H	Syntrophomonadaceae, Synergistaceae, Comamonadaceae, Rhodocyclaceae, Caldatribacteriaceae, Moraxellaceae, Hungateiclostridiaceae, Synergistaceae, Peptostreptococcales‐Tissierellales, Anaerolineaceae, and Bacteroidetes_vadinHA17	Desulfovibrionaceae
	H.HFV	H	Syntrophomonadaceae, Synergistaceae, Comamonadaceae, Caldatribacteriaceae, Moraxellaceae, Synergistaceae, Peptostreptococcales‐Tissierellales, Anaerolineaceae, Bacteroidetes_vadinHA17, and Anaerolineaceae	Streptococcaceae, Oscillospiraceae, Lachnospiraceae, Leuconostocaceae, and Monoglobaceae
Genus	H	C	Ahniella Brachymonas_	–
	H.LFV	H	Syntrophomonas Acetomicrobium Proteiniphilum Macellibacteroides, Candidatus_Caldatribacterium Ahniella Acinetobacter, and Brachymonas Fastidiosipila Keratinibaculum Lactibrio,	–
	H.HFV	H	Syntrophomonas Acetomicrobium Macellibacteroides Keratinibaculum Lactibrio	Oscillibacter Streptococcus Bilophila GCA–900066575
Species	H	C	Brachymonas_denitrificans	–
	H.LFV	H	Brachymonas_denitrificans and bacterium_enrichment_culture_clone_DPF35	–
	H.HFV	H	Brachymonas_denitrificans and bacterium_enrichment_culture_clone_DPF35	Lactobacillus_murinus, salivarius_subsp_thermophilus, Lactococcus_lactis, and Weissella_cibaria

The linear discriminant analysis effect size (LEfSe) is an analytical tool for discovering and interpretating high‐dimensional data biomarkers (genes, pathways, and taxons) which can be used to compare two or more groups and emphasizes statistical significance and biological correlation. It can find biomarkers with statistical difference between groups (Segata et al., 2011). Figure 6d–f shows the biomarkers with statistical differences among the groups. There were two species at the phylum level, five species at the class level, eight species at the order level, seven species at the family level, six species at the genus level, and two species at the species level which could be used as biomarkers. It indicated that the intake of fruits and vegetables could modulate the composition of gut microbiota, and this modulation effect was consistent with the changing trend in gut microbiota at the phylum level.

## 4 DISCUSSION

In recent years, many studies have shown that dietary supplements are a potentially viable nutritional strategy for the prevention of various diseases (Do et al., 2021). Vegetables and fruits eaten in daily life account for a large part of the diet and are the main source of nutrition. In this study, we found that fruits and vegetables can provide the body with more dietary fiber and achieve the effect of fat loss and weight loss, but no significant improvement in blood lipid levels was detected, which may be related to our increased intake of vegetables and fruits lower related. Veeramani selected 25 and 50 mg/kg BW of green vegetables to study their protective effect on fatty liver accumulation and oxidative damage induced by a high‐fat diet and found that 50 mg/kg BW dose of green vegetables had the greatest activity (Veeramani et al., 2017; Zhang et al., 2020). But there are also opposite results, adding 1% (w/w) kumquat fruit extract to a high‐fat diet can significantly reduce blood lipid levels and achieve the purpose of preventing and improving obesity and obesity‐related metabolic disorders (Tan et al., 2014). In addition to cellulose, vegetables and fruits also contain a variety of natural phenols, flavonoids, terpenes, and other substances, which have active functions such as antioxidant, anticancer, anti‐inflammatory, antibacterial, and antiaging (Kim et al., 2018; Rodríguez García & Raghavan, 2021; Sarma et al., 2016). For example, flavonols (kaempferol and quercetin) in onions, apples, and citrus fruits are involved in deglycosylation and stage metabolism in the small intestine. The gut microbiota produces deglycosylation, dihydroxylation, and cyclic cleavage, which converts them into phenolic acids involved in anti‐inflammatory, antioxidant, antiviral, and antibacterial effects (Barnes et al., 2011; Martinez et al., 2016).

In this study, the cellulose content was mainly used as the investigation condition. According to the different proportions of vegetable and fruit powder in the feed, it was found that high‐dose vegetable and fruit additions could enhance the antioxidant capacity of the liver of mice, improve fat accumulation, and increase the content of TC and TBA in the feces., which is consistent with the findings of Tong et al. (2015). The gut–liver axis is the two‐way communication between the gut and its microbes and the liver, influenced by diet, genetics, and the environment, and through the bile ducts, portal vein, and systemic circulation. Under normal circumstances, the intestinal barrier can effectively prevent intestinal microorganisms and harmful substances produced by their metabolism from entering the liver. When the intestinal barrier is damaged, the harmful substances in the intestine will invade the liver and participate in the occurrence and development of liver diseases. Disturbance of the gut–liver axis plays an important role in the development of liver disease. This study found that a high‐fat diet led to an increase in secondary bile acids and their metabolites and a decrease in short‐chain fatty acids in the gut by inducing alterations in gut microbial composition. The content of fecal bile acids in the group supplemented with fruit and vegetable powder was significantly increased, and the increase in fecal bile acids could reduce the possibility of obesity and hepatic steatosis. In addition, fruit and vegetable extracts have been shown to reduce obesity and alter gut microbial communities in high‐fat diet‐fed mice (Wu et al., 2019; Xu et al., 2019). The alpha‐diversity results in the study suggest that supplementation with vegetable and fruit powders can alleviate the severe changes in gut microbiota structure and composition induced by a high‐fat diet. The F/B ratio of the H group was higher than that of the C group, and the F/B ratio of the H.LFV and H.HFV groups was lower than that of the H group, indicating that the intake of vegetables and fruits can improve the mild inflammation caused by the increase in energy absorption. The mechanism is that Firmicutes are more efficient at extracting energy from food than Bacteroidetes, thereby promoting calorie absorption and leading to various diseases such as obesity (Li et al., 2019), therefore, an increase in the F/B ratio can lead to As a marker of obesity and dysbiosis (Han et al., 2021; Ke et al., 2020). Targeting the gut–liver axis is an effective way to address the mechanism of action of diet and related metabolites. We speculate that this will be a relatively novel direction and will be discussed more in the future.

## 5 CONCLUSION

In summary, our research shows that continuous intake of a certain amount of fruits and vegetables can significantly inhibit the increase in physical fitness, normalize the liver lipid accumulation in mice with metabolic syndrome, and reduce the risk of metabolic diseases; increase C57BL/TC and TBA excretion levels in feces of 6N mice; and improve liver antioxidant levels in C57BL/6N mice. In addition, fruit and vegetable intake not only promoted the abundance of Firmicutes but also inhibited Bacteroidetes, thereby increasing the diversity of gut microbiota in mice. Therefore, fruits and vegetables have a good prebiotic effect in the regulation of intestinal flora, and have the potential to prevent and improve metabolic syndrome and other related diseases.
